# Evaluating the impact of biodiversity offsetting on native vegetation

**DOI:** 10.1111/gcb.16801

**Published:** 2023-06-10

**Authors:** Sophus O. S. E. zu Ermgassen, Katie Devenish, B. Alexander Simmons, Ascelin Gordon, Julia P. G. Jones, Martine Maron, Henrike Schulte to Bühne, Roshan Sharma, Laura J. Sonter, Niels Strange, Michelle Ward, Joseph W. Bull

**Affiliations:** ^1^ Department of Biology University of Oxford Oxford UK; ^2^ Durrell Institute of Conservation and Ecology, School of Anthropology and Conservation University of Kent Canterbury UK; ^3^ School of Natural Sciences, College of Environmental Science and Engineering Bangor University Bangor UK; ^4^ Tampa Bay Estuary Program St. Petersburg Florida USA; ^5^ School of Global Urban and Social Studies RMIT University Melbourne Victoria Australia; ^6^ The University of Queensland, School of Earth & Environmental Sciences, and Centre for Biodiversity and Conservation Science Brisbane Queensland Australia; ^7^ Institute of Zoology Zoological Society of London London UK; ^8^ Department of Food and Resource Economics University of Copenhagen Copenhagen Denmark; ^9^ WWF—Australia Brisbane Queensland Australia

**Keywords:** Australian native vegetation, biodiversity offsets, counterfactual analysis, environmental policy, impact evaluation, net gain, no net loss, regulatory markets, statistical matching

## Abstract

Biodiversity offsetting is a globally influential policy mechanism for reconciling trade‐offs between development and biodiversity loss. However, there is little robust evidence of its effectiveness. We evaluated the outcomes of a jurisdictional offsetting policy (Victoria, Australia). Offsets under Victoria's Native Vegetation Framework (2002–2013) aimed to prevent loss and degradation of remnant vegetation, and generate gains in vegetation extent and quality. We categorised offsets into those with near‐complete baseline woody vegetation cover (“avoided loss”, 2702 ha) and with incomplete cover (“regeneration”, 501 ha), and evaluated impacts on woody vegetation extent from 2008 to 2018. We used two approaches to estimate the counterfactual. First, we used statistical matching on biophysical covariates: a common approach in conservation impact evaluation, but which risks ignoring potentially important psychosocial confounders. Second, we compared changes in offsets with changes in sites that were not offsets for the study duration but were later enrolled as offsets, to partially account for self‐selection bias (where landholders enrolling land may have shared characteristics affecting how they manage land). Matching on biophysical covariates, we estimated that regeneration offsets increased woody vegetation extent by 1.9%–3.6%/year more than non‐offset sites (138–180 ha from 2008 to 2018) but this effect weakened with the second approach (0.3%–1.9%/year more than non‐offset sites; 19–97 ha from 2008 to 2018) and disappeared when a single outlier land parcel was removed. Neither approach detected any impact of avoided loss offsets. We cannot conclusively demonstrate whether the policy goal of ‘net gain’ (NG) was achieved because of data limitations. However, given our evidence that the majority of increases in woody vegetation extent were not additional (would have happened without the scheme), a NG outcome seems unlikely. The results highlight the importance of considering self‐selection bias in the design and evaluation of regulatory biodiversity offsetting policy, and the challenges of conducting robust impact evaluations of jurisdictional biodiversity offsetting policies.

## Graphical abstract

We evaluate the outcomes of one of the world's oldest biodiversity offsetting systems in Victoria (Australia), comparing changes in offset woody vegetation cover from 2008 to 2018 with two sets of controls derived from alternative approaches to estimating the counterfactual. We identify concerns about the additionality of offsets. Our results suggest offsets increased vegetation cover by 0–180 ha over a 10‐year period. The limited publicly available data on clearances associated with offsets suggests the policy did not achieve its goal of a net gain in vegetation extent and quality, although a lack of data on vegetation condition means this result is not conclusive.

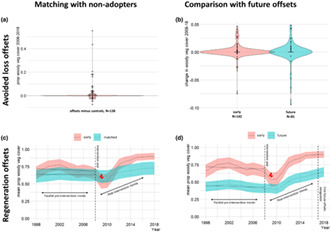

## 1 INTRODUCTION

Land use change associated with resource consumption is the main global driver of biodiversity and ecosystem service loss (Marques et al., 2019; Maxwell et al., 2016). A range of policy instruments have emerged to attempt to govern potential trade‐offs between land use change and biodiversity protection (Bull et al., 2020; zu Ermgassen, Utamiputri, et al. 2019). Among the most influential is biodiversity offsetting, which is being applied in a growing number of jurisdictions globally, as well as under major multilateral banks' biodiversity safeguard policies (Bull & Strange, 2018; zu Ermgassen, Utamiputri, et al. 2019). The purpose of biodiversity offsetting is to achieve no net loss or net gain (NNL/NG) of biodiversity resulting from development impacts, following the application of the rest of the mitigation hierarchy (avoid; minimise; restore; offset; Bull et al., 2013). Offsetting has also received much attention in national and international policy discussions for its perceived promise as a scalable mechanism for attracting private finance into addressing global shortfalls in biodiversity funding (Deutz et al., 2020).

### 1.1 The evidence underpinning biodiversity offsetting

Despite the increasing uptake and high‐level interest in policy and financial circles, there is a substantial shortfall in robust evidence supporting its effectiveness at achieving NNL of biodiversity. Offsetting has been critiqued on economic (Spash, 2015), political (Walker et al., 2009), ecological (Maron et al., 2012; Moreno‐Mateos et al., 2015), ethical (Apostolopoulou & Adams, 2017) and governance grounds (Damiens et al., 2021), so high‐quality evidence demonstrating its effectiveness should be required to justify its widespread adoption. Recent reviews of offsetting outcomes suggest offsets achieve better outcomes in less ecologically complex or faster‐maturing habitats such as some types of wetlands, and worse outcomes in slow‐maturing habitats such as woodlands (Josefsson et al., 2021; Theis et al., 2020; zu Ermgassen, Baker, et al. 2019). However, these reviews highlight that existing evidence is sparse, and commonly relies on weak study designs.

Robust impact evaluation methods are increasingly being applied in other domains of conservation science such deforestation policy (Simmons et al., 2018). Quasi‐experimental designs improve the evaluation of impacts compared with traditional between‐group comparisons because they generate more credible counterfactuals against which to assess the ‘true’ impact of policy (Maron et al., 2013; Schleicher et al., 2020). So far, just three studies have used quasi‐experimental approaches to evaluate the outcomes of biodiversity compensation policies. Sonter et al. (2019) used statistical matching coupled with a means comparison to evaluate the effect of Californian species conservation banks (most of which do not have an explicit NNL objective) on land use change, and showed these banks perversely averted considerable gains in natural habitats relative to counterfactual sites. Inkinen et al. (2022) evaluated the outcomes of US compensatory wetland mitigation from 1995 to 2000 using a difference‐in‐differences framework with data on mitigation banks and those that were planned but not implemented, and they found that although most gains in wetland area were additional, the policy as a whole led to losses in wetland averaging 1600 acres/year. Devenish et al. (2022) evaluated the impact of the Ambatovy mine's offsets in Madagascar using matching and fixed effects regression, and showed that the associated offset is on track to successfully achieve NNL of forest because it likely would prevent as much deforestation as the mine caused.

The preliminary promising result of Devenish et al. (2022) cannot be generalised to jurisdictional offsetting systems (offsetting systems embedded in national or regional policy, often associated with regulatory markets). The Ambatovy offset was a single well‐resourced, private sector‐led offset (in part implemented to satisfy biodiversity safeguards of large multilateral lenders) in a context of substantial background deforestation, which featured a bespoke biodiversity loss/gain calculation method suited to the local context. Jurisdictional offsetting policies function differently from direct voluntary offsets (Koh et al., 2019). They are typically implemented as part of the planning process, applying standardised and simplified biodiversity assessment methods across a wide variety of ecological contexts, and these policies are designed to satisfy multiple, sometimes conflicting objectives, such as streamlining planning processes whilst simultaneously achieving biodiversity outcomes (Damiens et al., 2020; Evans, 2017; zu Ermgassen et al., 2021). For example, streamlining planning often leads to measuring biodiversity using simplified assessment tools which are easily applied by consultants, leading to these systems delivering nature improvements designed to optimise the delivery of the specific biodiversity features chosen in policy, potentially driving homogenisation of habitats (Lave et al., 2010; Rampling et al., 2023; Tillman & Matthews, 2023). These and other governance challenges such as capacity shortages in regulators have led to systemic implementation failures (Bezombes et al., 2019; Evans, 2017, 2023; Samuel, 2020).

One major, yet underexplored, challenge to jurisdictional offset systems is self‐selection bias. Offsets can only be additional and therefore legitimate compensation for biodiversity losses elsewhere if they induce conservation actions that result in gains that would not have happened in the absence of the offset transaction (Maron et al., 2013). Central to the market‐like logic behind offsets is that sellers would not have implemented conservation in the absence of their offsetting payment. However, previous qualitative work with offset‐adopters demonstrates that there are various motivations for implementing offsets on private land, many of which are non‐financial and tied to landowners values and attitudes towards nature (Brown et al., 2021; Groce & Cook, 2022; Selinske et al., 2016, 2022). This indicates there is a risk of self‐selection bias in offsetting systems, with programmes enrolling landholders who might already have been implementing nature‐friendly management practices or are less likely to clear existing native vegetation on their land, thereby undermining the additionality of receiving offsetting payments. This is a well‐recognised issue in conservation incentive schemes such as payment for ecosystem services (Jack & Jayachandran, 2019).

The most robust evaluation of a jurisdictional native vegetation offsetting policy to date is Gibbons et al. (2018). They evaluated the outcomes of the New South Wales (Australia) offsetting system, which is predominantly based on ‘avoided loss’ (i.e. an offset system that compensates for biodiversity losses by reducing the threats to existing habitats so future losses are avoided). Gibbons et al. estimated that it will take 146 years for existing offsets to avoid as much deforestation as that caused by the associated developments. The estimated counterfactual deforestation rate (i.e. what would have happened in the absence of the intervention) is based on the long‐term deforestation rate for the region, which leaves opportunities to improve on the methodology to generate more context‐specific counterfactuals by using quasi‐experimental methods.

Impact evaluations require the estimation of a counterfactual against which to measure the impacts of the intervention. In practice, there can be multiple justifiable counterfactuals for a given conservation intervention (Bull et al., 2021) with widely differing effects on policy outcomes (Sonter et al., 2017). Statistical matching is one widely used method for identifying appropriate controls, based on minimising the differences in covariates known to be confounders (i.e. to be associated both with exposure to the treatment and the outcome of interest) between the treated and untreated samples (Schleicher et al., 2020; Stuart, 2010). Many conservation impact evaluation studies base their matching on a set of biophysical and spatially explicit economic covariates such as topography or access to roads. However, a key predictor of the implementation of conservation actions on private land is the psychosocial characteristics of the landowners themselves (Brown et al., 2021; Simmons et al., 2021). Spatially explicit data on these attributes are often absent or prohibitively costly to collect so they tend not to be included in matching studies. Failing to capture these attributes might lead to biased results and there is increasing attention in the conservation science literature surrounding the importance of non‐random treatment assignment (Jones et al., 2022; Rasolofoson, 2022).

### 1.2 Victoria's Native Vegetation Framework

Australia has lost one third of all its native vegetation since European settlement. The state of Victoria introduced the Native Vegetation Framework (hereafter ‘the Framework’) in 2002 with an overall goal of ‘A reversal, across the entire landscape, of the long‐term decline in the extent and quality of native vegetation, leading to a net gain’ (Department of Sustainability and Environment, 2002). One of the environmental measures introduced by the Framework was native vegetation offsetting, whereby clearing of native vegetation was to be compensated through conservation actions aimed at improving the extent and/or condition of native vegetation elsewhere (Supporting Information). Over the following years, the government created a regulatory market for offsets whereby land clearers could purchase credits to offset their native vegetation liabilities. The first offsets implemented under the Framework entered the system in 2006. Offset agreements last 10 years (i.e. commit landowners to 10 years of implementing management measures) with sites then theoretically protected in perpetuity without management thereafter (but see Damiens et al., 2021). The policy goal and gain scoring methods were altered in 2013 then 2017 following regulatory reform, although the core principles and loss/gain scoring methods remain similar and relevant today.

Here, we evaluate the impacts of completed, 10‐year‐old offsets implemented within the native vegetation offsetting system in Victoria, Australia, one of the oldest jurisdictional offsetting policies in the world. We use a robust counterfactual‐based design to evaluate whether, and to what extent, Victoria's first tranche of offsets under the Framework resulted in improvements (or reduced losses) in the extent of native vegetation relative to control sites.

## 2 METHODOLOGY

Under the Framework, offsets were considered to generate four types of biodiversity gain (the sum of which can be sold as credits): ‘prior management gain’, ‘security gain’, ‘maintenance gain’ and ‘improvement gain’ (DSE, 2006; Table 1). The metric used to quantify losses and gains is ‘habitat hectares’ (Parkes et al., 2003): a composite indicator combining habitat area with condition. Condition is measured by comparing the value of a range of ecological attributes with those of intact reference sites for the same habitat type (Supporting Information). If offsets are effective, these gains collectively should mean smaller reductions in woodland extent and condition and greater increases in woodland extent or condition, than would otherwise have occurred.

**TABLE 1:** Summary of the different categories of biodiversity gains achievable according to the Native Vegetation Framework (DSE, 2006) and their impact on observable outcomes.

Gain category	Explanation	Avoid condition losses	Generate condition gains	Avoid losses in area of vegetation	Increase vegetation area
Prior management	Landholders are awarded units as incentive to participate in the scheme (is not associated with any ecological gains)				
Security	Landholders implement legal mechanisms to protect native vegetation from anthropogenic conversion (e.g. enter into management agreement)	✓		✓	
Maintenance	Landholders implement management measures to maintain the current condition of native vegetation over time (e.g. invasive plant removal, stock control)	✓		✓	
Improvement	Landholders implement management measures to improve the condition or extent of native vegetation over time (e.g. active planting)		✓		✓

We could not conclusively evaluate whether the framework delivered NG as there are no publicly available data on the vegetation clearance events that were enabled through selling the native vegetation offsets in our dataset. Additionally, vegetation condition as measured using habitat hectares is based on site‐based attributes that can only be assessed through site visits (e.g. ground flora, dead wood) and cannot be effectively captured via satellite data without comprehensive model testing and validation against site‐based data (which is rarely publicly‐available). However, we could evaluate whether and to what extent woody native vegetation area—a key component of habitat hectares—was influenced at offset sites through gains from prevented losses and increases. Gains additional to those achieved at otherwise‐similar sites would indicate at least partial compensation for losses elsewhere. We used satellite data on vegetation cover to estimate additional gains in native vegetation extent occurring at offset sites between 2006 and 2018.

We used two alternative approaches to estimate the counterfactuals. First, we compared native vegetation outcomes in offset sites registered between 2006 and 2008 (hereafter “early offsets”) with land parcels not used as offsets (“non‐adopters”) that were matched on biophysical and spatial economic variables (Schleicher et al., 2020). We refer to this as our “matched” set of controls. For the second approach, we compared native vegetation outcomes observed on these early offsets with those on sites which were not offsets for the duration of our evaluation, but were registered as offsets at the end of our evaluation's time series (2017–2019; hereafter “future offsets”). This set of controls therefore comprises land parcels that were not matched on biophysical covariates, but where landholder psychosocial characteristics were more likely to be similar as landholders opted into delivering offsets in both cases (Simmons et al., 2021).

### 2.1 Data preparation

We obtained shapefiles of all offsets registered on the Native Vegetation Offsets Register from 2002 to 2019 from the Victorian Department for Environment, Land, Water and Planning (DELWP). The database captured 398 offset land parcels across 67 different land holdings for the years included in our analysis (2006–2008, 2017–2019) covering a total of 5377 ha. To match offset land parcels with land parcels not under offset management, we used state‐wide land use maps for 2006 (coincident with the system's first registered offsets) which included the spatial boundary and land use information for every land parcel in the state of Victoria (DELWP, 2022).

Our woody vegetation cover outcome dataset was derived from the National Forest and Sparse Woody Vegetation Data produced by the Australian government, a Landsat‐derived raster with 25 m pixel spatial resolution (Department of the Environment and Energy, 2019). Cells were classified into three categories: no woody vegetation, sparse woody vegetation (5%–19% canopy cover) and complete woody vegetation (>20% canopy cover, vegetation >2 m tall) for an annual time series from 1998 to 2018. Positional accuracy was estimated at 10 m. While offsets in Victoria span various habitat types including grasslands, we restricted our analysis only to offsets containing ecological vegetation classes which, when in good condition, would be expected to be classified as complete woody vegetation cover (i.e. good condition examples of this ecological vegetation class are >2 m tall and with >20% canopy cover in each pixel; Supporting Information). Our outcome variable was the proportion of the total number of pixels in each offset/land parcel classified as complete woody vegetation cover in each year, calculated in QGIS (version 3.20.3). Pixels intersecting with the boundary of the polygon were removed from the analysis. We excluded offsets smaller than 10 pixels (6250 m^2^; *n* = 15 early offsets, *n* = 105 among the future offsets) as the proportion of vegetation cover was sensitive to small changes in these parcels.

For our statistical matching we used a suite of geographical predictors both theoretically and empirically linked with forest loss/gain in multiple contexts (Table S3; Eklund et al., 2016; Geldmann et al., 2019; Negret et al., 2020; Simmons et al., 2018; Sonter et al., 2019) and which might have affected exposure to the treatment (i.e. whether land was enrolled as an offset). Our predictors of agricultural opportunity costs and ecological productivity (which determine agricultural profitability and are therefore likely to affect treatment assignment) included mean rainfall, slope, elevation, temperature, soil water, soil carbon, and baseline woody vegetation cover; predictors of human pressure and accessibility include remoteness, distance from roads and distance from the nearest protected area. Other important geographical variables included the land use of each land parcel in 2006, and X and Y coordinates of the parcel centroid. Given the offsets predominantly mapped onto agriculture, forestry, and conservation area land uses in the state‐wide land use dataset, we restricted our potential matched controls to landholdings from these three land use types. We also collated data on all bushfires detected from 2008 to 2018 from Ward et al. (2019). The fire data were used to test whether the evaluation results are explained by differences in burning between offsets and controls. Information about data sources is in the Supporting Information (Table S3).

### 2.2 Analytical approach

The distribution of our outcome variable (proportion woody vegetation cover) at baseline across our offset sites was skewed: many offset sites had proportion woodland cover at or approaching 1 (i.e. the upper bound of our outcome variable) in our evaluation's baseline year (2008; Figure S1). We subset the data into two main categories of offsets—offsets focusing predominantly on avoiding losses of native vegetation and maintaining or improving vegetation condition that had a proportion baseline woody vegetation cover greater than .95 (henceforth “*avoided loss*” offsets, *N* early = 142, *N* future = 81); and those aiming to achieve both maintaining native vegetation cover and condition and increases in cover and condition, whose proportion woody vegetation cover in the baseline year 2008 was less than .95 (henceforth “*regeneration*” offsets, *N* early offsets = 54, *N* future offsets = 121). We chose .95 as the threshold for our core analysis as it retained a sufficient sample size for the statistical analysis of both pools of offsets (i.e. lower thresholds substantially reduced the sample size for regeneration offsets, e.g. threshold .9, *N* early offsets = 37). We varied this assumption and evaluated the impact on our results as a robustness check. We analysed both sets of offsets separately. This analytical approach matched important features of the policy: avoided loss offsets act through different mechanisms and often different management regimes from offsets targeting improvements in woody vegetation cover (Table 1), and including both within the same regression framework would constrain our ability to evaluate the effectiveness of the various mechanisms and management measures underpinning the different offset types.

**FIGURE 1:**
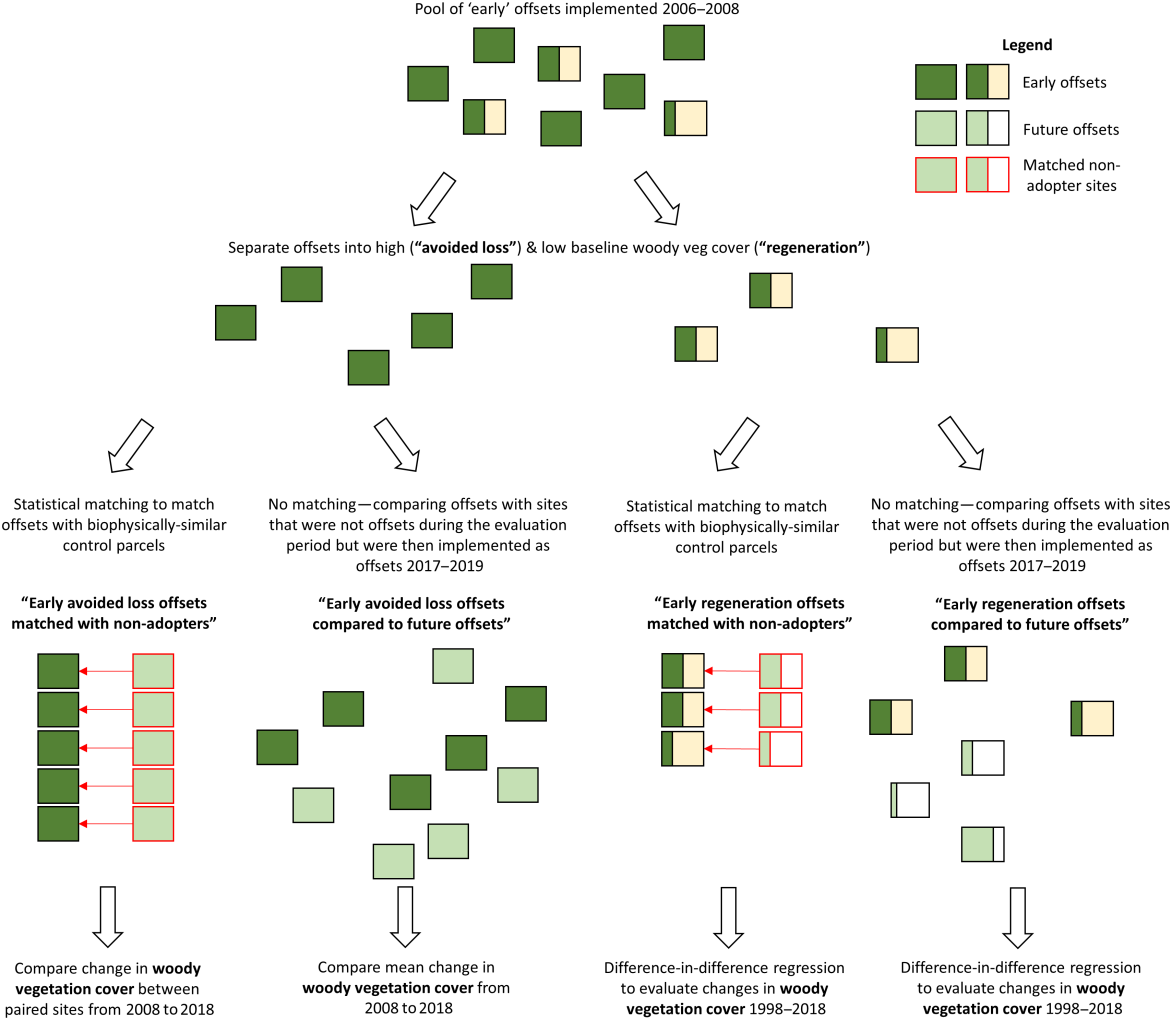
An overview of our methodological approach.

For both of these sets of offsets, we then compared outcomes with those observed in control land parcels selected using our two alternative approaches to estimating the counterfactual (i.e. comparing early offsets with matched non‐adopters, and future offsets; Figure 1). The advantages and disadvantages of the two approaches to estimating the counterfactual are given in Table 2.

**TABLE 2:** The advantages and disadvantages of both of our approaches to estimating the counterfactuals used in this study.

	Matching with non‐adopters	Comparing with future offsets
Advantages	Control parcels were selected to be as similar as possible to offsets according to numerous biophysical and land cover covariates known to affect changes in woody vegetation cover and to influence exposure to the treatment	Control land parcels would be more likely to be managed by landholders who possess similar psychosocial characteristics to landholders in the offsets sample. This approach therefore potentially reduces self‐selection bias. There is evidence of self‐selection bias documented by qualitative studies into the drivers of conservation on private land in Australia (Selinske et al., 2016, 2022)
Disadvantages	No data were available on the owners of land parcels, who may have different psychosocial characteristics between offset and control samples (a potentially important confounder). The risk of self‐selection bias could lead to an overestimate of the treatment effect	Compared to the matched sample, the control parcels were less similar to the offsets with regards to biophysical covariates, so multiple factors could be explaining differences in outcomes between offsets and counterfactuals Land ownership and economic incentives may have changed during the time series, and landholders implementing early offsets will not be a perfect psychosocial match for those implementing future offsets, so self‐selection bias is only partly accounted for

### 2.3 Statistical matching

We used statistical matching to generate one of our two samples of control land parcels (Schleicher et al., 2020; Stuart, 2010). We ran the matching separately for both our regeneration and avoided loss subsets. Our pool of potential control parcels was every land parcel in the state after filtering out parcels which did not fall within the size range or land use categories of our offsets (*N* = 364,290). We restricted the categorical variable land use to exact matches. We implemented matching in R using the MatchIt package (Ho et al., 2011). Following the protocol of Schleicher et al. (2020), we ran alternative matching specifications (Mahalanobis distance matching, propensity score matching, varying calipers) without replacement and inspected the results for evidence of differences in performance using Love plots (Supporting Information) before selecting the approach to use in the analysis.

### 2.4 Statistical analyses

#### 2.4.1 Evaluating differences in woody vegetation cover between avoided loss offsets and controls

We compared changes in woody vegetation cover from 2008 to 2018 in the early avoided loss offsets with that in our two sets of control land parcel samples (i.e. future offsets, and matched non‐adopters). Inspection of both early and future avoided loss offsets revealed that 70% of sites lost no woodland cover in any of the years along our time series (2008–2018). We ran exploratory linear regressions comparing the change in woody vegetation cover from 2008 to 2018 across offsets and controls using the economic and biophysical covariates used in matching as covariates. These regressions found no significant relationships between whether the site was an offset or control and changes in woody vegetation cover 2008–2018, but these regressions had little explanatory power (e.g. regression comparing changes in woody vegetation cover between early avoided loss offsets and future offsets had an adjusted *R* ^2^ = .098), and diagnostic plots showed non‐normality in the distribution of residuals. This was expected as linear regression assumes that the outcome variable is unbounded, whereas most of our observations lay on the bound (i.e. most avoided loss offsets had a baseline woody vegetation cover of 1).

Therefore, we resorted to conducting a simple comparison of the mean change in woody vegetation cover from 2008 to 2018 between our early offsets and both sets of controls. When comparing changes in woody vegetation cover between early offsets and future offsets, we compared the sample means using a two‐tailed Wilcoxon rank sum test. To evaluate the impacts of offsets compared to matched non‐adopter controls, we conducted a two‐tailed Mann–Whitney test comparing changes in woody vegetation cover between each paired offset and control. If the means comparisons found a significant difference between woody vegetation loss between offsets and controls, we multiplied the difference in woody vegetation loss between offsets and controls by the total area of avoided loss offsets in our sample to estimate the total area of vegetation saved from clearance by offsets.

#### 2.4.2 Regeneration offsets

##### Evaluating changes in woody vegetation cover in offsets relative to controls

The baseline woody vegetation cover of our regeneration offsets was not at the upper bound of our outcome variable, so we analysed the effects of offsets on changes in woody vegetation cover using linear mixed effects models. Linear models are the most commonly‐used methods for evaluating the effectiveness of land management on continuous parcel‐level land cover outcomes, even when the outcome variable is bounded (Archibald, 2020; Jones & Lewis, 2015; Nolte et al., 2019). To evaluate the effectiveness of regeneration offsets, we implemented the generalised difference‐in‐difference framework developed in Wauchope et al. (2021) on our complete set of offsets and control land parcels, running separate regressions for offsets and each of the two sets of controls. The core regression framework could be expressed as

$$\begin{array}{c} \mathrm{veg}\;{\text{cover}}_{t,i}=\beta_{0}+\beta_{1}{\mathrm{BA}}_{t}+\beta_{2}{\mathrm{CI}}_{i}+\beta_{3}T_{t}+\beta_{4}{\mathrm{BA}}_{t}{\mathrm{CI}}_{i}+\beta_{5}{\mathrm{BA}}_{t}T_{t}\\ \;+\beta_{6}{\mathrm{CI}}_{i}T_{t}+\beta_{7}{\mathrm{BA}}_{t}{\mathrm{CI}}_{i}T_{t}+\beta X_{t,i}+\left(1|k\right)+\varepsilon_{t,i}, \end{array}$$

where ‘veg cover’ is our outcome variable given at time step *t* for offset or control land parcel *i*, BA is a dummy variable representing whether the observation is before or after the offset implementation date, CI is a dummy representing whether the time series belongs to the control or offset sample, *T* is the year of the observation centred around the intervention year, and ***X*** represents a vector of covariates for each land parcel, and *k* represents the overall landholding ID. The coefficient of interest was the interaction term *β*_7_, which represents the difference in the change in trend in forest cover after the offset implementation between the offset and control parcels. Theoretically, for regeneration offsets, the change in woody vegetation cover over time should be more positive after the offset is implemented than before (meaning woody vegetation cover is increasing at a faster rate), and this before‐after change should be greater than in the control (given the lack of an intervention). Further information about the meanings of other coefficients is given in Wauchope et al. (2021); none were of direct interest to our research question.

We set our intervention date at 2008 and therefore implicitly grouped together all early offsets implemented from 2006 to 2008. The assumption that all offsets are implemented in 2008 would be problematic if we were interested in the immediate change in woodland cover resulting from the intervention (*β*_5_), but we were only interested in the long‐term change in trend. Time lags between changes in management and woody vegetation growth mean that we would expect little change in woodland coverage caused by changes in management in offset sites to occur immediately (i.e. between 2006 and 2008), so we lost little relevant information contributing to the change in long‐run trend from this assumption.

To account for repeated observations, heteroscedasticity and non‐normality, we used a linear mixed effects model with landholding ID as a random effect, subtracting the mean for each covariate from all covariates to ensure model convergence and using the lme4 package in R version 4.4.1 (Bates et al., 2014; Wauchope et al., 2019). In addition to the biophysical covariates mentioned above, we included X and Y coordinates as covariates to partially address spatial autocorrelation, and included the baseline proportion woodland cover and its square to account for nonlinear relationships between baseline cover and subsequent changes in cover over time (Love, 2022). We checked for collinearity between variables using the ‘corpcor’ package (Schafer et al., 2017), and found three variables (y‐coordinates, elevation, temperature) with correlation in excess of .75, and evaluated the effect of dropping these variables on our results. Diagnostic plots show there remains some residual heteroskedasticity, but linear mixed effects models are robust to violations of the distributional assumptions (Schielzeth et al., 2020). We compared the model performance to an alternative specification where we used a linear model without random effects, comparing model performance based on AICs.

Difference‐in‐difference analyses rely on the assumption of parallel trends between the intervention and counterfactual sites in the period before the intervention (i.e. 1998–2008), which we tested for through visual inspections and by regressing the pre‐intervention woody vegetation cover data against the interaction between whether the site is from the control or intervention sample, and year (Devenish et al., 2022). If the interaction is significant, it implies that there is a significantly different time trend between the offsets and controls.

The coefficient *β*_7_ can be interpreted as the relative change in woody vegetation cover in each year that offsets deliver above that delivered by controls following the date of offset implementation. Therefore, to estimate the total area of woody vegetation gain attributable to the implementation of regeneration offsets in each year, we multiplied the total area of regeneration offsets in our sample by *β*_7_. To estimate the total change in woody vegetation across the whole 10‐year lifetime of these offsets, we multiplied this by 10.

##### Evaluating the effect of excluding sites burned in wildfires

Wildfires have the potential to bias our results if by chance they impact either offsets or controls differently, as woody vegetation can recover quickly after fire in a way which is not attributable to the treatment (offset management). This is especially of concern in Victoria during our evaluation period, as the Black Saturday fires in 2009 burned approximately 450,000 ha of bushland. To identify offsets and controls potentially impacted by wildfires, we visually assessed the time‐series woody vegetation cover data for each land parcel for unusual reductions in woodland cover, then cross‐referenced the parcel location against spatial data on all bushfires in Victoria occurring from 2008 to 2018 as assembled in Ward et al. (2019). We identified one land holding containing 12 early offsets which burned completely during the Black Saturday fires. We reran our core analyses excluding the affected land parcel.

##### Evaluating sensitivity of the results to the threshold between avoided loss and regeneration offsets

To evaluate the effect of the choice of threshold (proportion woody vegetation cover above/below .95) used for classifying offsets into the two offset categories, we reran our core analyses at alternative threshold levels (.9 and .8) and summarised the effects on the results.

##### Evaluating the effects of local spillovers

To test for local spillovers whereby land conversion was displaced from the offsets into the surrounding landscape inflating the rate of habitat loss (Ferraro et al., 2019), we assessed whether any of the matched land parcels fell within 500 m of offset sites, and if so, we reran our outcome regressions excluding all these matched land parcels which fell within 500 m of the offset sites and investigated the effects on our coefficient of interest. We recognise that detecting spillovers in this analysis is unlikely, given the change in vegetation extent caused by offsets is negligible relative to the size of the study area.

## 3 RESULTS

### 3.1 Dataset summary

Our study spanned 196 early offsets (total area of 3203 ha), 364,290 non‐adopter land parcels from which to select controls, and 202 future offsets, that is those that became offsets between 2017 and 2019 (total area 2174 ha). On average, early offsets had higher levels of baseline woodland cover, were larger, and were located in different local government areas from the future offsets established from 2017 to 2019. The Supporting Information contains details of the distribution of covariates between the samples (Figure S1) and the geographical distribution of early‐ and future offsets (Figure S2).

### 3.2 Avoided loss offsets

#### 3.2.1 Comparison of early avoided loss offsets and matched non‐adopters

For avoided loss offsets, our best‐performing matching specification (Supporting Information) was nearest neighbour matching based on Mahalanobis distances and a caliper of .25 standard deviations. This specification found matches for 138/142 early offsets with standardised mean differences below .25 for all covariates.

**FIGURE 2:**
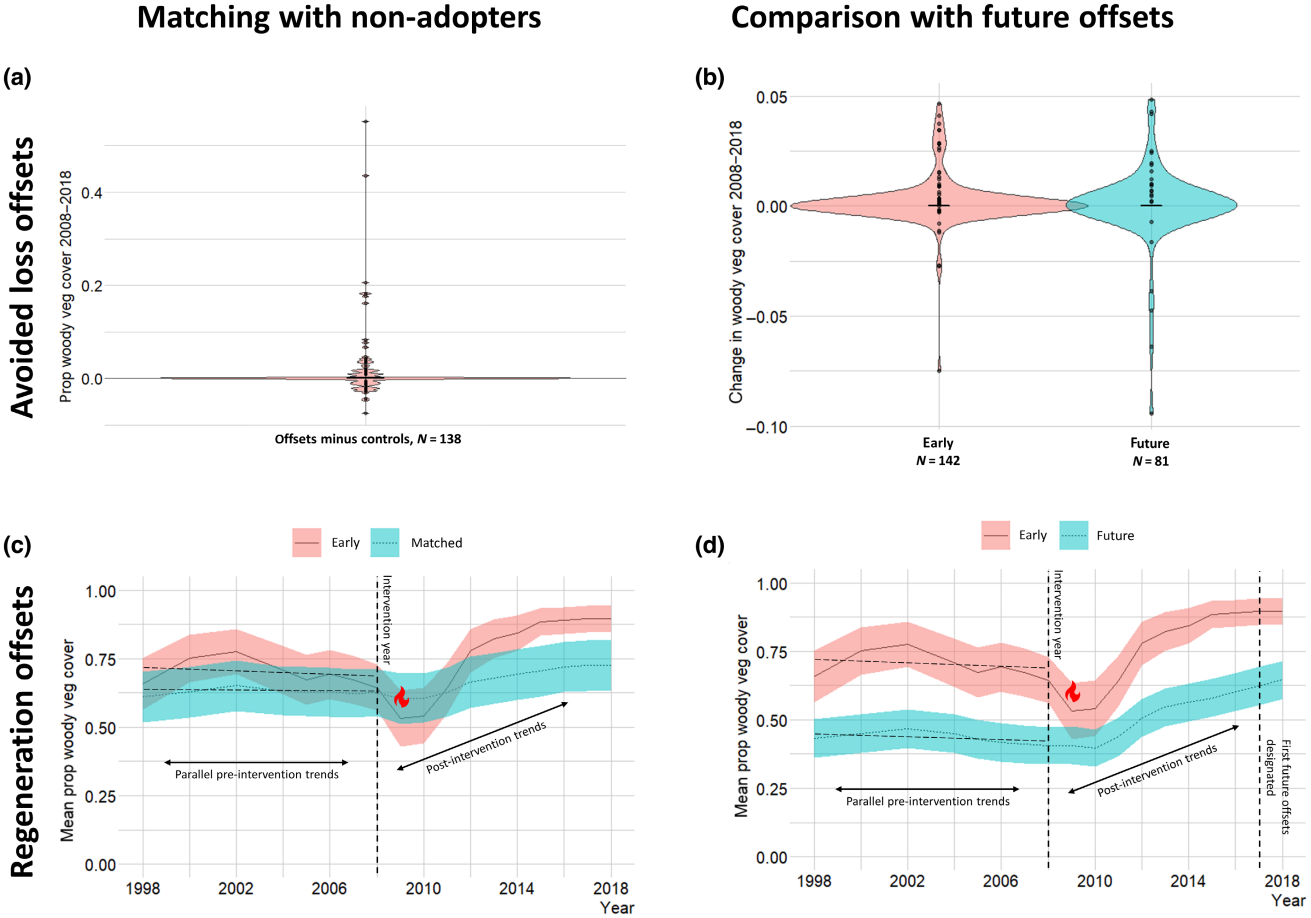
Visual summary of the results of the evaluation comparing woody vegetation cover between offsets and controls. (a) Combined violin and boxplot of the difference in the change in the proportion of woody vegetation cover from 2008 to 2018 between early offsets and matched non‐adopter control pairs. Positive differences indicate offsets which outperformed their paired controls, negative differences indicate paired controls which outperformed their offsets. (b) Combined violin and boxplot of the change in the proportion of woody vegetation cover from 2008 to 2018 in early offsets compared to future offsets. (c) Changes in the mean proportion of sites covered with woody vegetation across regeneration offsets and matched non‐adopter controls from 1998 to 2018. Error bars represent 95% confidence intervals for each year. The flame symbol in 2009 marks the Black Saturday fires, which severely burned one landholding containing 12 offsets. Hatched lines before 2008 represent the pre‐intervention trends in the change in woody vegetation cover in the offsets and control sites. Arrows explain the key component of the analysis, comparing the change in trend before and after 2008 between offsets and controls. (d) Changes in the mean proportion of sites covered with woody vegetation across regeneration offsets and future offsets from 1998 to 2018. Error bars represent 95% confidence intervals for each year. Vertical line in 2017 marks the first future offsets entering Victoria's native vegetation offsetting system.

On average there was no impact of avoided loss offsets on woody vegetation change detected using this first approach to estimating the counterfactual. The mean change in the proportion of woody vegetation cover in sites for which matches were identified was +.002 in offsets and −.013 in matched non‐adopter controls (no significant difference between paired offsets and controls at the 5% significance level, Mann–Whitney test, *p* = .09; Figure 2a). Looking at individual pairs of early offsets and their matched controls, there was no difference in woody vegetation change for 65 pairs (47% of pairs), and controls outperformed offsets for 31 pairs (22% of pairs). In 42 pairs (30% of pairs), offsets outperformed controls (and for seven pairs, offsets prevented the loss of >10% woody vegetation cover relative to their matched controls).

#### 3.2.2 Comparison of early avoided loss offsets and future offsets

There was no impact detected of avoided loss offsets using the second approach to estimating the counterfactual (comparing woody vegetation change in early offsets and sites that were not offsets but would go on to be designated as offsets at the end of the study's time series). There was no clear difference in the change in woody vegetation cover in early avoided loss offsets compared to future offsets (early mean woody vegetation change = .002, future offsets = .001, Wilcoxon rank sum test *p* = .99; Figure 2b), indicating that offsets protecting existing woody vegetation did not avoid more woodland loss than controls.

### 3.3 Regeneration offsets

#### 3.3.1 Comparison of early regeneration offsets and matched non‐adopters

For regeneration offsets, our best‐performing matching specification was 1:1 nearest‐neighbour Mahalanobis distance matching and a caliper of .25 standard deviations. The standardised mean difference was successfully reduced below .25 for all covariates and below .1 for 8/13 covariates, indicating high‐quality matches (Supporting Information; Figure S3). 53/54 offsets from this subset were successfully matched with control land parcels. Our test for parallel trends in pre‐intervention rates of woodland change held (Supporting Information), so we proceeded with the difference‐in‐difference analysis.

Using this first approach to approximating the counterfactual, we estimated that woody vegetation cover increased in regeneration offsets by, on average, 2.75% (CI 1.9%–3.6%) more each year than in counterfactual land parcels post‐intervention (Figure 2c). This is based on the regression, with parcel ID as a random effect, which yielded a parameter of interest *β*_7_ = .0275, *p* = <.001, CI = .019–.036 with a model *R* ^2^ = .72 (full regression output in Supporting Information). This model also is associated with a lower AIC than our alternative model specifications. Given the area of regeneration offsets was 501 ha, this implies that over the 10‐year offset management period regeneration offsets led to an additional ~138 ha (CI 95–180 ha) increase in woody vegetation cover relative to the counterfactual. However, this result is sensitive to the exclusion of the landholding containing 12 early offsets which was burned completely in the 2009 Black Saturday fires. When this site was excluded from the sample, our estimate for *β*_7_ changed to .024, implying offsets resulted in a 120 ha increase in woody vegetation relative to the counterfactual (CI 78–163 ha).

#### 3.3.2 Comparison of early regeneration offsets and future offsets

For regeneration offsets using our second approach to approximating the counterfactual, our test for parallel trends in the pre‐treatment period between early and future regeneration offsets found no significant difference in trends (Supporting Information), justifying our subsequent difference‐in‐difference analysis.

Using this second approach to estimating the counterfactual suggests that woody vegetation in early regeneration offsets increased by, on average, 1.5% more each year than in future offsets. This estimate is smaller than that obtained using the matching approach described above. Under our core model, our parameter of interest (the coefficient for our interaction terms, *β*_7_) was significant at the conventional .05 significance level (*β*_7_ = .0147, *p* = .004, CI = .0037–.0193, model *R* ^2^ = .80). This model outperformed alternative specifications according to AIC values. This implies that over the 10‐year offset management period, regeneration offsets led to 74 ha (CI 19–97 ha) of additional woody vegetation (Figure 2d). However, this result disappears when excluding the landholding containing the 12 early offsets which burnt during the 2009 Black Saturday fires (*β*_7_ = .006, *p* = .12), implying that offsets caused no increase in woody vegetation relative to the counterfactual (CI −8 to 70 ha).

#### 3.3.3 Sensitivity of results to varying the threshold between avoided loss and regeneration offsets

Varying the threshold (proportion of the site covered by woody vegetation) used to categorise offsets into the avoided loss or regeneration categories had some impact on the magnitude of the impacts of regeneration offsets and avoided loss offsets. The general pattern was for the effect size of regeneration offsets to rise as the threshold fell, whilst this did not impact the outcomes of avoided loss offsets. However, this also led to the classification of fewer offsets (therefore a smaller overall area) as regeneration offsets, which led to a reduction in the total area identified as additional despite the increase in effect size (Supporting Information). The avoided loss outcomes were unaffected, with neither means comparison yielding a clear difference between offsets and controls.

### 3.4 Comparisons of gains with losses

**FIGURE 3:**
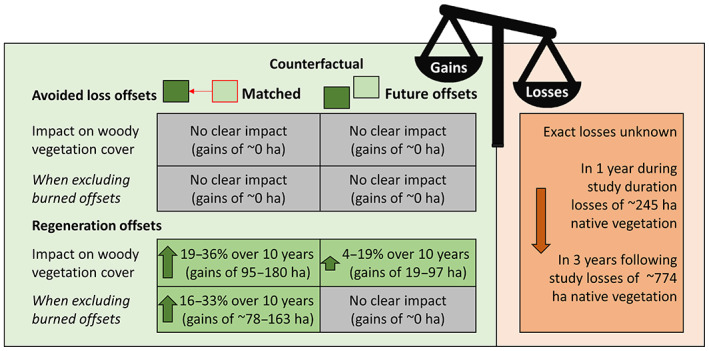
Visual summary of the outcomes of each component of the impact evaluation (and whether the evidence suggests that gains in woody vegetation were generated by the policy), and a comparison with estimated losses under the policy (from limited publicly available information).

To know whether the offsets enrolled under the framework contributed effectively to delivery of NGs in woody vegetation under the framework we would need information on the area of losses that were incurred under the framework for which this offsets were used as compensation. This is difficult to obtain. One government document reports that 245 ha of native vegetation were cleared under Victoria's native vegetation policy in the year July 2006–August 2007 (Parkes, 2007). Another document suggests that in 3 years (2008–2011), 774 ha of native vegetation were permitted to be cleared (DSE, 2012). Our results suggested that avoided loss offsets had no clear impact on woody vegetation cover (i.e. they protected and enhanced the quality of vegetation that would not have been cleared in the absence of offset management), and regeneration offsets led to a 0%–3.6% per year increase in native vegetation cover relative to controls (range representing results from our range of alternative regression specifications), or a total additional increase of 0–180 ha of woody vegetation from 2008 to 2018. This additional increase is smaller than the known area of losses for a single year out of the 3 years‐worth of offsets included in our evaluation (i.e. offsets allocated from 2006 to 2008). This suggests that it is unlikely, given reasonable assumptions, that NNL or NG of woody vegetation was delivered under this policy, although no information is available on what proportion of native vegetation clearance corresponded to ‘woody’ vegetation which is our analysis's outcome variable (Figure 3).

## 4 DISCUSSION

Our study finds evidence that biodiversity offsets under Victoria's Native Vegetation Framework delivered limited additionality and therefore had a limited impact on woody vegetation extent, and suggest that most losses of native vegetation cover were not counterbalanced. The results varied somewhat dependent on which approach was used to estimate the counterfactual. When we matched offsets to non‐adopter land parcels which were biophysically similar, the results suggested that regeneration offsets at least may have delivered greater increases in woody vegetation cover. However, this approach does not account for differences in the landholder characteristics which may be important confounders, as both likely affect the chance of land being enrolled as well as the management of that land. When instead we compared early offsets with future offsets (thereby potentially partially capturing self‐selection bias, but which were not matched on biophysical variables, Supporting Information), this estimated impact on woody vegetation extent weakened (and disappeared when one landholding containing 12 offsets which burned in the Black Saturday fires was excluded). However, we are not able to conclusively determine whether this was due to the presence of self‐selection bias, the prospect of enrolling in offset management affecting landholder vegetation clearance behaviour in the lead‐up to enrolling, or because of differences in biophysical variables between offsets and future offsets. However, the self‐selection bias theory matches qualitative research conducted in Australian offset systems (Selinske et al., 2016, 2022).

### 4.1 Challenges to determining whether jurisdictional offset systems achieve their ecological goals

This evaluation does not allow us to draw firm conclusions about whether offsets under the Framework achieved their policy goal of NG of habitat hectares (i.e. did gains associated with offsets effectively counterbalance losses) for a number of reasons. First, data limitations meant we were not able to capture impacts on native vegetation condition, a key component of habitat hectares. However, our results would mean that NGs in native vegetation as measured by habitat hectares could only be achieved through large increases in vegetation condition, but vegetation condition gains that come from management such as that done in offset sites are likely to be small (Dorrough et al., 2019; Oliver et al., 2021). Second, to know if NG has been achieved, information on the losses (habitat hectares of native vegetation cleared for which the offsets were compensation) is needed. Little such information is available and the limited information we have is not disaggregated by habitat type; so while we can estimate gains in extent of native woody vegetation, the only information we have on loss relates to native vegetation including non‐woody types, such as grassland. Third, we evaluate the outcomes of the offsetting system for the duration of the 10‐year management contracts. However, in theory, offsets in the Victorian system are protected in perpetuity (but see Damiens et al., 2021). Therefore, over longer timescales, the offsets in our sample may accrue gains via avoided losses that were not detectable over the time series of this evaluation (though given there was no gains through avoided loss over 10 years this seems unlikely to be significant; and the policy assumes that all gains accrue over the 10‐year management period). Fourth, we only evaluate the outcomes of offsets for which changes in woody vegetation cover is a suitable outcome variable, so our results do not reflect on the outcomes of some common offset types such as grassland offsets.

Biodiversity offsetting systems continue to be rolled out globally (zu Ermgassen, Utamiputri, et al. 2019), yet, knowing if they are actually helping achieve this is deeply challenging. Offsetting systems around the world consistently fail to achieve basic criteria for enabling a robust understanding of their actual impacts (Kujala et al., 2022). Ongoing monitoring of biodiversity offsets to ensure they are still performing their required ecological function is frequently neglected, and much information about the performance of offsets is not publicly available (Bull et al., 2018). Many of these barriers affected our analysis. In Victoria, under the Framework, there were multiple assessment pathways for offsets depending on the magnitude of the initial clearing event, with only larger and more ecologically significant impacts (approximately one‐third of all impacts) and their offsets recorded by the state government. There was therefore no information available on the number or size of clearance events and offsets administered by councils. The lack of publicly available information about changes in the condition of offset sites was a major barrier to evaluation, given that condition is a key determinant of a site's habitat hectares score. While offset owners do have to submit annual monitoring reports to DELWP, these do not require field assessments to assess how the condition score of site changes over time. We therefore have little information about how responsive condition scores are to change, even under idealised offset management, which means it is challenging to conclusively know whether offset management is improving the condition of sites. Many of these barriers remain in today's reformed offsetting policy, with a recent Auditor General's report making numerous recommendations for how to improve the implementation, monitoring, data management and compliance surrounding Victoria's offsetting policy (VAGO, 2022). Our results should be taken in the context of that independent report, which found that native vegetation is unambiguously declining in extent and condition across the state (VAGO, 2022), primarily because of illegal vegetation clearance for which no compensation is occurring. Various other problems with the offsetting system were identified, including the risk of overallocation of credits from established offsets, and serious governance shortfalls for offsets regulated by councils (which fall under a different referral pathway from the offsets evaluated in this paper).

### 4.2 Implications for offsetting policies

Our analysis has several important implications for the design and implementation of offset polices. Firstly, the evaluation indicates that offsets protecting existing areas with high levels of woody vegetation cover did little to protect woody vegetation from clearance, invalidating some of the mechanisms through which security gain and maintenance gain generate credits in Victoria's offsetting system, and threatening the core logic of ‘avoided loss’‐based offset systems (Maseyk et al., 2020). This provides further empirical evidence supporting the already extensive literature showing that the offset multipliers used in offset policies are much lower than the true multipliers required to achieve NNL (Bull et al., 2017; Laitila et al., 2014). A clear implication is the need to increase the size of biodiversity offsetting multipliers used in jurisdictional policies to reflect the true gain from avoided losses, if they are to credibly claim NNL outcomes within a reasonable timeframe.

Our work re‐affirms the value of robust study designs and time‐series data in impact evaluations. Vegetation cover increased in offsets across our time‐series, and simple before‐after designs would have unambiguously demonstrated that offsets have increased woody vegetation cover. However, the comparison of changes in vegetation against carefully‐chosen controls shows that much of this vegetation enhancement would have also have occurred in the absence of offset management, as vegetation across Victoria recovered following the end of the Millennium drought in 2010. Our analysis adds to the literature highlighting the necessity of applying more robust study designs in conservation science to develop an improved understanding of conservation effectiveness (Christie et al., 2019; Ferraro & Hanauer, 2014; Wauchope et al., 2022).

Our study also contributes to the evidence‐base showing that ecological gains from avoided losses are consistently revealed to have been overestimated by ex‐post impact evaluations, joining a range of recent studies which have identified critical additionality shortcomings in forest‐based carbon and biodiversity offsetting systems in Australia and elsewhere (Badgley et al., 2022; Gibbons et al., 2018; Macintosh et al., 2022; West et al., 2023). Under both the Framework and Victoria's contemporary offsetting system, landholders are allocated more credits for native vegetation retention than vegetation regeneration (as revegetation activities do not accrue credits from prior management gain, and accrue fewer credits for vegetation maintenance and improvement; DSE, 2006). Whilst this is justified on the grounds that restored ecosystems have lower levels of ecological function and higher risk of establishment than existing vegetation, revegetation has a substantially higher probability of additionality which is implicitly neglected in the gain scoring methodology. Increasing the incentives for revegetation relative to the maintenance of existing vegetation could help secure more additional offsetting gains.

Perhaps the most novel contribution of this study is the way the results are consistent with the theory of self‐selection bias potentially reducing the additionality of gains supposedly generated within offsetting regulatory markets. Our evidence for this is the smaller effect sizes we found when estimating the counterfactual by comparing early offsets to future offsets, rather than comparing early offsets with non‐adopter parcels matched on biophysical and spatial economic covariates (with no attempt to account of psychosocial characteristics of landholders). This evidence of course has important limitations. Our within‐sample approach to overcoming self‐selection bias assumes that landholders who opted into offsets in the early and future time‐periods share characteristics that affect their entry into the programme and land management practices. Additionally, we are unable to evaluate whether or not landholders pre‐emptively altered their vegetation management practices in the run‐up to enrolling their sites as offsets, given that intact vegetation has the potential to generate more credits than cleared sites. We do not have the data to evaluate the magnitude of these effects. However, our self‐selection bias hypothesis fits with the patterns in our data, the qualitative data from elsewhere in Australia indicating that many landholders enrol in conservation management because they have pro‐environmental or land stewardship attitudes (Selinske et al., 2016, 2022), quantitative data showing that vegetation clearance behaviours are partially explained by landholders' psychosocial traits (Brown et al., 2021; Simmons et al., 2021), and empirical work exploring the implications of self‐selection bias in related Payment for Ecosystem Services schemes (Jack & Jayachandran, 2019). Ultimately, future work may be able to more rigorously demonstrate self‐selection bias by collecting data on landholders' psychosocial traits and modelling their propensity to participate in the offsetting programme as a function of their psychosocial traits (e.g. Archibald, 2020; Simmons et al., 2021), then comparing ecological outcomes between participant and non‐participant landholders matched on psychosocial traits alongside biophysical covariates.

If we accept that our study design might partially capture the effect of self‐selection bias, and that this explains some of the difference in outcomes between our two evaluation approaches, then there would be important implications. Offsetting regulatory markets all over the world select offset sites through a process of voluntary landholder enrolment (Koh et al., 2019), and the gain‐scoring methods used to quantify the number of biodiversity credits generated commonly rely on static (e.g. England's Biodiversity Net Gain; zu Ermgassen et al., 2021) or declining (e.g. Victoria, New South Wales; Maseyk et al., 2020) counterfactuals. If a proportion of offsets are delivering gains that largely would have been delivered anyway (i.e. they protect habitat that would not otherwise be under much threat, or lead to biodiversity recovery that would have occurred regardless), this undermines the value of jurisdictional offset systems as a mechanism for reconciling development and nature objectives. Jurisdictional offset policies conserve biodiversity through two key mechanisms: (1) they aim to make up for the harm caused by the development project; and (2) they aim to internalise the price of biodiversity loss into the development process, disincentivising damage to areas of high biodiversity in the first place (Calvet et al., 2015; Pascoe et al., 2019; zu Ermgassen et al., 2020). If the additionality of offsetting actions is questionable, then this partially undermines the first theory of change, and alters the benefits of offsets to more closely resemble those of a direct tax on biodiversity loss, with revenues directed towards agricultural subsidies. We therefore highlight how to overcome selection bias in offsetting systems as a promising and important area for future research, with opportunities for learning from other ecological incentive mechanisms such as Payment for Ecosystem Services (Jack & Jayachandran, 2019).

## 5 CONCLUSION

Biodiversity compensation systems are being increasingly adopted around the world (zu Ermgassen, Utamiputri, et al. 2019). Offsets are also widely perceived in policy and business circles as a key solution for addressing the global biodiversity finance gap. But offsets are not pure conservation funds—they are defensive expenditures (Spash, 2015) as each offset is associated with a loss of biodiversity elsewhere. Therefore, each offset that fails to deliver the expected biodiversity outcomes results in harm that goes uncompensated, contributing to further biodiversity declines. In many jurisdictions, biodiversity offsetting regulatory markets have become sizeable industries with their own interests and growth‐agendas, estimated at a global market value of $6–9 bn (Deutz et al., 2020). But a focus on the market size and financial performance of biodiversity offsetting fundamentally misidentifies their core policy goal—to deliver as a minimum NNL of biodiversity (zu Ermgassen et al., 2020). This evaluation found that it is unlikely that offsetting under Victoria's Native Vegetation Framework achieved its policy goal of NG in habitat hectares. The implications are clear. This study highlights there is an urgent need for: improved transparency in offsetting systems, to allow for rigorous evaluations of their contributions to biodiversity conservation; serious reflection in the policymaking community on the relative benefits of avoided loss versus revegetation‐focused offsets; and further research addressing the challenge of self‐selection bias in offsets on private land, so that offsets can deliver on their potential as parts of the policy mix for addressing global biodiversity funding shortfalls and the ecological impacts of new development.

## CONFLICT OF INTEREST STATEMENT

The authors declare that they have no conflicts of interest affecting this work.

## Supporting information


Appendix S1.

## Data Availability

Data subject to third party restrictions. The data that support the findings of this study are available from DELWP. Restrictions apply to the availability of these data, which were used under license for this study. De‐identified data from offset and matched land parcels has been archived at https://osf.io/hbvt8/.
