# Supplementary material for: Evaluating the impact of biodiversity offsetting on native vegetation
Source: Glob Chang Biol. 2023 Jun 10;29(15):4397–411. doi: 10.1111/gcb.16801 (PMC10946555; doi:10.1111/gcb.16801)
Supplement: Supplementary file 1 — Appendix S1. [file GCB-29-4397-s001.pdf]

Supporting information for “Evaluating the impact of biodiversity offsetting on native vegetation”

Supplementary Introduction

Victoria’s Native Vegetation Framework

The Native Vegetation Framework ran in Victoria from 2002-2013, when it was superseded by new native vegetation regulation associated with a slightly-altered offset policy. Under the Framework, applications to remove native vegetation were sent to local councils and processed through the planning system, with larger impacts and those to ecologically significant biodiversity conventionally referred to the state authorities for approval (this pathway comprised approximately one third of applications in 2010/2011; DSE 2012). Offsets required to compensate for clearance events that were referred to the State government were then registered.

Entering into an offset agreement committed landholders to both protect the registered native vegetation in perpetuity, and implement management actions (most commonly grazing exclusion and invasive plant or weed removal) under a 10-year management plan to deliver enhancements in biodiversity across that time period. Biodiversity gains were calculated using the ‘habitat hectares’ (HH) currency (Parkes et al. 2003), which allowed an estimate of the predicted gains in biodiversity over the 10-year management lifetime. These biodiversity gains translated into biodiversity credits which could be used directly to compensate for native vegetation clearance conducted by the same entity as that creating the offset (‘first party offset’), or sold to other land clearers to offset their liabilities (‘third party’). The State government implemented the Bushbroker programme, an initiative to create a regulatory market in offsets whereby land clearers could purchase offset credits to offset their native vegetation liabilities, which has since developed into a fully-fledged state offsetting sector brokered predominantly by private firms.

Habitat hectares

The HH approach is one of the original and most influential area*condition biodiversity metrics implemented in biodiversity offsetting systems around the world, which has served to underpin numerous derivative metrics such as England’s Biodiversity Metric (Crosher et al. 2019). To calculate a site’s HH score, a qualified consultant conducts a site-based assessment, and scores the ecological quality of each ecological vegetation class (EVC) on the impacted site according to a number of ecological criteria (Table S1; Parkes et al. 2003). Each criteria is scaled so an EVC scores the maximum number of points if the ecological criteria are equivalent to those found at an intact reference patch of that EVC. Different ecological criteria contribute variably to the overall habitat score for the site, with the most ecologically important criteria contributing more to the overall habitat score. The total habitat score for the site adds up to a maximum of 100. This habitat score is then multiplied by the total area of the site in ha to yield the HH score. For example, 10ha of an intact reference EVC would score 10HH, and 10ha of a moderate condition EVC which achieved an overall habitat score of 50 would yield 5HH.

Table S1. Components of the Habitat Hectares score. Adopted from Parkes et al. (2003)

Ecological criteria	Maximum value (sums to 100)
Coverage of large trees	10
Canopy cover	5
Richness and degree of modifications of understory strata	25
Invasiveness and coverage of weeds	15
Plant recruitment	10
Coverage of organic litter	5
Total length of logs on site	5
EVC patch size	10
Neighbourhood / connectivity with surrounding vegetation patches	10
Distance to core area (vegetation patch >50ha)	5

Supplementary Methodology

Criteria for including offsets in evaluation

To decide which EVCs to include in our analysis, we used the information from the EVC benchmarks (https://www.environment.vic.gov.au/biodiversity/bioregions-and-evc-benchmarks). We include all EVCs which, when in good condition, would be expected exceed the threshold of >2m vegetation height and >20% canopy cover based on the information provided in the EVC benchmarks (Table S2).

Table S2. Summary of all of the EVCs included in offsets in the Victorian offset database, noting which would be expected to be classified as complete woody vegetation cover in our outcome dataset and therefore which are included in our evaluation

IBRA bioregion	EVC	Include in analysis
Central Victorian Uplands	Lowland Forest	yes
Central Victorian Uplands	Heathy Dry Forest	yes
Central Victorian Uplands	Grassy Dry Forest	yes
Central Victorian Uplands	Herb-rich Foothill Forest	yes
Central Victorian Uplands	Valley Grassy Forest	yes
Central Victorian Uplands	Heathy Woodland	yes
Central Victorian Uplands	Plains Grassy Woodland	yes
Central Victorian Uplands	Box Ironbark Forest	yes
Central Victorian Uplands	Rocky Chenopod Woodland	yes
Central Victorian Uplands	Hills Herb-rich Woodland	yes
Central Victorian Uplands	Grassy Woodland	yes
Dundas Tablelands	Damp Sands Herb-rich Woodland	yes
Dundas Tablelands	Plains Grassy Woodland	yes
East Gippsland Lowlands	Coast Banksia Woodland	yes
East Gippsland Lowlands	Damp Sands Herb-rich Woodland	yes
East Gippsland Lowlands	Banksia Woodland	yes
East Gippsland Lowlands	Lowland Forest	yes
East Gippsland Lowlands	Estuarine scrub	yes
East Gippsland Uplands	Dry Valley Forest	yes
East Gippsland Uplands	Grassy Woodland	yes
East Gippsland Uplands	Lowland Herb-rich Forest	yes
Greater Grampians	Grassy Dry Forest	yes
Greater Grampians	Valley Grassy Forest	yes
Greater Grampians	Heathy Woodland	yes

Gippsland Plain	Coast Banksia Woodland	yes
Gippsland Plain	Damp Sands Herb-rich Woodland	yes
Gippsland Plain	Sand Heathland	no
Gippsland Plain	Wet Heathland	no
Gippsland Plain	Coastal Saltmarsh	no
Gippsland Plain	Estuarine Wetland	no
Gippsland Plain	Lowland Forest	yes
Gippsland Plain	Riparian Forest	yes
Gippsland Plain	Herb-rich Foothill Forest	yes
Gippsland Plain	Damp Forest	yes
Gippsland Plain	Valley Grassy Forest	yes
Gippsland Plain	Heathy Woodland	yes
Gippsland Plain	Swamp Scrub	no
Gippsland Plain	Plains Grassy Woodland	yes
Gippsland Plain	Swampy Riparian Woodland	yes
Gippsland Plain	Plains Grassy Wetland	no
Gippsland Plain	Sand Forest	yes
Gippsland Plain	Sedge Wetland	no
Gippsland Plain	Mangrove Shrubland	no
Gippsland Plain	Plains Grassy Forest	yes
Gippsland Plain	Creekline Herb-rich Woodland	yes
Gippsland Plain	Grassy Woodland	yes
Gippsland Plain	Sedgy Swamp Woodland	yes
Gippsland Plain	Damp Heathy Woodland	yes
Gippsland Plain	Coastal Alkaline Scrub	yes
Gippsland Plain	Estuarine Flats Grassland	no
Gippsland Plain	Swampy Woodland	yes
Gippsland Plain	Estuarine scrub	yes
Glenelg Plain	Coastal Headland Scrub	no
Glenelg Plain	Coastal Mallee Scrub	yes
Goldfields	Grassy Dry Forest	yes
Goldfields	Herb-rich Foothill Forest	yes
Goldfields	Heathy Dry Forest	yes
Goldfields	Valley Grassy Forest	yes
Goldfields	Heathy Woodland	yes
Goldfields	Plains Grassy Woodland	yes
Goldfields	Box Ironbark Forest	yes
Goldfields	Alluvial Terraces Herb-rich Woodland	yes
Goldfields	Creekline Grassy Woodland	yes
Goldfields	Hillcrest Herb-rich Woodland	yes
Goldfields	Hills Herb-rich Woodland	yes
Goldfields	Sandstone Ridge Shrubland	yes
Goldfields	Plains Grassy Wetland	no
Goldfields	Grassy Woodland	yes
Goldfields	Plains Woodland	yes
Highlands - Northern Fall	Heathy Dry Forest	yes
Highlands - Northern Fall	Shrubby Dry Forest	yes
Highlands - Northern Fall	Grassy Dry Forest	yes
Highlands - Northern Fall	Herb-rich Foothill Forest	yes
Highlands - Northern Fall	Rocky Outcrop Shrubland	no
Highlands - Northern Fall	Damp Forest	yes
Highlands - Northern Fall	Montane Grassy Woodland	yes
Highlands - Northern Fall	Montane Riparian Woodland	yes
Highlands - Northern Fall	Swampy Riparian Woodland	yes
Highlands - Southern Fall	Lowland Forest	yes
Highlands - Southern Fall	Riparian Forest	yes
Highlands - Southern Fall	Heathy Dry Forest	yes
Highlands - Southern Fall	Shrubby Dry Forest	yes
Highlands - Southern Fall	Grassy Dry Forest	yes
Highlands - Southern Fall	Herb-rich Foothill Forest	yes
Highlands - Southern Fall	Damp Forest	yes
Highlands - Southern Fall	Wet Forest	yes
Highlands - Southern Fall	Shrubby Foothill Forest	yes

Highlands - Southern Fall	Valley Grassy Forest	yes
Highlands - Southern Fall	Riparian Thicket	yes
Highlands - Southern Fall	Box Ironbark Forest	yes
Highlands - Southern Fall	Creekline Grassy Woodland	yes
Highlands - Southern Fall	Swampy Riparian Woodland	yes
Highlands - Southern Fall	Valley Heathy Forest	yes
Highlands - Southern Fall	Grassy Forest	yes
Highlands - Southern Fall	Plains Grassy Forest	yes
Highlands - Southern Fall	Creekline Herb-rich Woodland	yes
Highlands - Southern Fall	Shrubby Damp Forest	yes
Highlands - Southern Fall	Damp Heathy Woodland	yes
Highlands - Southern Fall	Gully Woodland	yes
Lowan Mallee	Lowan Sands Mallee	yes
Lowan Mallee	Heathy Mallee	yes
Lowan Mallee	Treed Sandstone Ridge Shrubland	yes
Murray Fans	Riverine Chenopod Woodland	yes
Murray Fans	Grassy Riverine Forest	yes
Murray Fans	Riverine Grassy Woodland	yes
Murray Fans	Plains Woodland	yes
Murray Fans	Riverine Swamp Forest	yes
Murray Fans	Riverine Swampy Woodland	yes
Murray Fans	Sedgy Riverine Forest	yes
Murray Fans	Lignum Swampy Woodland	yes
Murray Mallee	Woorinen Sands Mallee	yes
Murray Mallee	Loamy Sands Mallee	yes
Murray Mallee	Chenopod Mallee	yes
Murray Mallee	Plains Woodland	yes
Murray Mallee	Woorinen Mallee	yes
Northern Inland Slopes	Heathy Dry Forest	yes
Northern Inland Slopes	Grassy Dry Forest	yes
Northern Inland Slopes	Herb-rich Foothill Forest	yes
Northern Inland Slopes	Valley Grassy Forest	yes
Northern Inland Slopes	Plains Grassy Woodland	yes
Northern Inland Slopes	Box Ironbark Forest	yes
Northern Inland Slopes	Alluvial Terraces Herb-rich Woodland	yes
Northern Inland Slopes	Creekline Grassy Woodland	yes
Northern Inland Slopes	Granitic Hills Woodland	yes
Northern Inland Slopes	Spring Soak Woodland	yes
Northern Inland Slopes	Low Rises Grassy Woodland	yes
Northern Inland Slopes	Shrubby Granitic-outwash Grassy Woodland	yes
Otway Plain	Coastal Saltmarsh	no
Otway Plain	Lowland Forest	yes
Otway Plain	Shrubby Dry Forest	yes
Otway Plain	Herb-rich Foothill Forest	yes
Otway Plain	Heathy Woodland	yes
Otway Plain	Swamp Scrub	yes
Otway Plain	Swampy Riparian Woodland	yes
Otway Plain	Plains Sedgy Wetland	yes
Otway Plain	Tall Marsh	no
Otway Plain	Plains Brackish Sedge Wetland	no
Otway Ranges	Wet Forest	yes
Otway Ranges	Cool Temperate Rainforest	yes
Otway Ranges	Shrubby Foothill Forest	yes
Otway Ranges	Shrubby Wet Forest	yes
Robinvale Plains	Riverine Chenopod Woodland	yes
Strzelecki Ranges	Lowland Forest	yes
Strzelecki Ranges	Damp Forest	yes
Strzelecki Ranges	Wet Forest	yes
Strzelecki Ranges	Cool Temperate Rainforest	yes
Strzelecki Ranges	Warm Temperate Rainforest	yes
Strzelecki Ranges	Shrubby Foothill Forest	yes
Strzelecki Ranges	Heathy Woodland	yes
Strzelecki Ranges	Swamp Scrub	yes

Strzelecki Ranges	Swampy Riparian Woodland	yes
Strzelecki Ranges	Riparian Scrub	yes
Victorian Alps	Montane Damp Forest	yes
Victorian Alps	Montane Riparian Thicket	yes
Victorian Alps	Sub-alpine Shrubland	no
Victorian Alps	Sub-alpine Woodland	yes
Victorian Alps	Sub-alpine Wet Heathland	no
Victorian Riverina	Plains Grassy Woodland	yes
Victorian Riverina	Floodplain Riparian Woodland	yes
Victorian Riverina	Ridged Plains Mallee	yes
Victorian Riverina	Riverine Chenopod Woodland	yes
Victorian Riverina	Plains Grassland	no
Victorian Riverina	Red Gum Swamp	yes
Victorian Riverina	Riverine Grassy Woodland	yes
Victorian Riverina	Plains Woodland	yes
Victorian Riverina	Riverine Swamp Forest	yes
Victorian Riverina	Riverine Swampy Woodland	yes
Victorian Riverina	Sedgy Riverine Forest	yes
Victorian Riverina	Lignum Swampy Woodland	yes
Victorian Riverina	Chenopod Grassland	no
Victorian Volcanic Plain	Lowland Forest	yes
Victorian Volcanic Plain	Herb-rich Foothill Forest	yes
Victorian Volcanic Plain	Swamp Scrub	yes
Victorian Volcanic Plain	Plains Grassy Woodland	yes
Victorian Volcanic Plain	Higher Rainfall Plains Grassy Woodland	yes
Victorian Volcanic Plain	Floodplain Riparian Woodland	yes
Victorian Volcanic Plain	Creekline Grassy Woodland	yes
Victorian Volcanic Plain	Lignum Swamp	yes
Victorian Volcanic Plain	Plains Grassy Wetland	no
Victorian Volcanic Plain	Plains Grassland	no
Victorian Volcanic Plain	Heavier-soils Plains Grassland	no
Victorian Volcanic Plain	Low-rainfall Plains Grassland	no
Victorian Volcanic Plain	Grassy Woodland	yes
Victorian Volcanic Plain	Stony Rises Woodland	yes
Victorian Volcanic Plain	Cane Grass Wetland	no
Victorian Volcanic Plain	Riparian Woodland	yes
Victorian Volcanic Plain	Plains Sedgy Wetland	no
Victorian Volcanic Plain	Stony Knoll Shrubland	yes
Victorian Volcanic Plain	Escarpment Shrubland	yes
Warrnambool Plain	Riparian Forest	yes
Warrnambool Plain	Herb-rich Foothill Forest	yes
Warrnambool Plain	Swamp Scrub	yes
Warrnambool Plain	Coastal Dune Scrub	no
Warrnambool Plain	Coastal Headland Scrub	no
Warrnambool Plain	Aquatic Herbland	no
Wimmera	Low Rises Woodland	yes
Wimmera	Lower Rainfall Shallow Sands Woodland	yes

Data sources

Table S3. Summary of the data layers used as covariates in the regressions and statistical matching, and justifications

Dataset	Details	Source
Outcome variables		
Woody vegetation cover	Landsat satellite imagery is used to estimate woody vegetation extent annually from 1998-2018. Each 25m^2 pixel can take on a value of 0 (no woody vegetation), 1 (sparse woody vegetation, canopy cover between 5-19%), or 2 (minimum 20% canopy cover, with vegetation >2 metres high and a minimum area of	https://data.gov.au/data/dataset/d734c65e-0e7b-4190-9aa5-ddbb5844e86d/resource/bf7420cc-2ec7-470d-87ba-f0a2c0ea1b60/download/woody-

	0.2 hectares. Sparse woody is defined as woody vegetation with a canopy cover between 5-19 per cent.	vegetation-extent-v3_0-metadata_2018.pdf
Agricultural opportunity cost / ecological variables		
Rainfall	Mean annual precipitation from 1981-2010, 5km resolution.	http://www.bom.gov.au/climate/data-services/maps.shtml
Elevation	Digital terrain model, 20m resolution.	https://www.land.vic.gov.au/maps-and-spatial/spatial-data/vicmap-catalogue/vicmap-elevation
Slope	Slope, 20m resolution. Obtained using the 'Slope' command in QGIS using 20m digital terrain model as input.	https://www.land.vic.gov.au/maps-and-spatial/spatial-data/vicmap-catalogue/vicmap-elevation
Temperature	Mean annual temperature from 1961-1990, 2.5km resolution.	http://www.bom.gov.au/climate/data-services/maps.shtml
Soil carbon	Soil carbon in top 5cm of soil, 3 arc second (~30m) resolution.	https://www.clw.csiro.au/aclep/soilandlandscapegrid/GetData-GIS.html
Soil water capacity	Soil water capacity in top 5cm of soil, 3 arc second (~30m) resolution.	https://www.clw.csiro.au/aclep/soilandlandscapegrid/GetData-GIS.html
Remoteness / human pressure variables		
Distance from roads	Distance to major roads in 2016. 100m resolution raster. Values represent the distance (in kilometres) from the cell centre to the road recorded in Open Street Map.	https://www.worldpop.org/geodata/summary?id=17302
Remoteness	1km resolution remoteness raster. "ARIA+ measures remoteness in terms of access along the road network from populated localities to each of five categories of Service Centre based on population size. If one thinks of ARIA as based on the distances people have to travel to obtain services, then populated localities are where they are coming from, and Service Centres are where they are going to." Remoteness ranges from 1-15.	https://arts.adelaide.edu.au/hugo-centre/services/aria
Distance from conservation areas	100m resolution raster of distance to nearest conservation area in 2006. All conservation areas in 2006 are marked with a specific numerical code (9*) in the Victorian 2006 land use dataset (below). These conservation areas were rasterised, and distance from areas was obtained using the proximity (raster distance) tool in QGIS.	See 'land use' below.
Other geographical variables		
IBRA subregions	IBRA 5.1 regions, the ecological regions used under the native vegetation framework framework (established in 2000). Polygons.	http://www.environment.gov.au/fed/catalog/search/resource/details.page?uuid=%7BA98C1395-42E9-43AE-9EE4-0083B0414658%7D
Land use	Spatial boundaries and land use for every cadastral land parcel in Victoria 2006, derived from the government's land use information system (polygon). Each parcel is marked with a specific numerical land use code, allowing the identification of all land parcels used for farming, nature conservation, forestry, and other land uses across the state.	https://discover.data.vic.gov.au/dataset/victorian-land-use-information-system-2006-2007<br>http://data.daff.gov.au/brs/data/warehouse/pe_abares99001806/GuidelinesLandUseMappingLowRes2011.pdf
Local government authorities (LGAs)	Spatial boundaries of LGAs and unincorporated Alpine resorts in Victoria, polygons.	https://www.land.vic.gov.au/maps-and-spatial/spatial-data/vicmap-catalogue/vicmap-admin
Burn scars from 2008 onwards	Collated data representing fire locations from Ward et al. (2019).	https://conbio.onlinelibrary.wiley.com/doi/full/10.1111/csp2.117

Supplementary Results

Dataset description

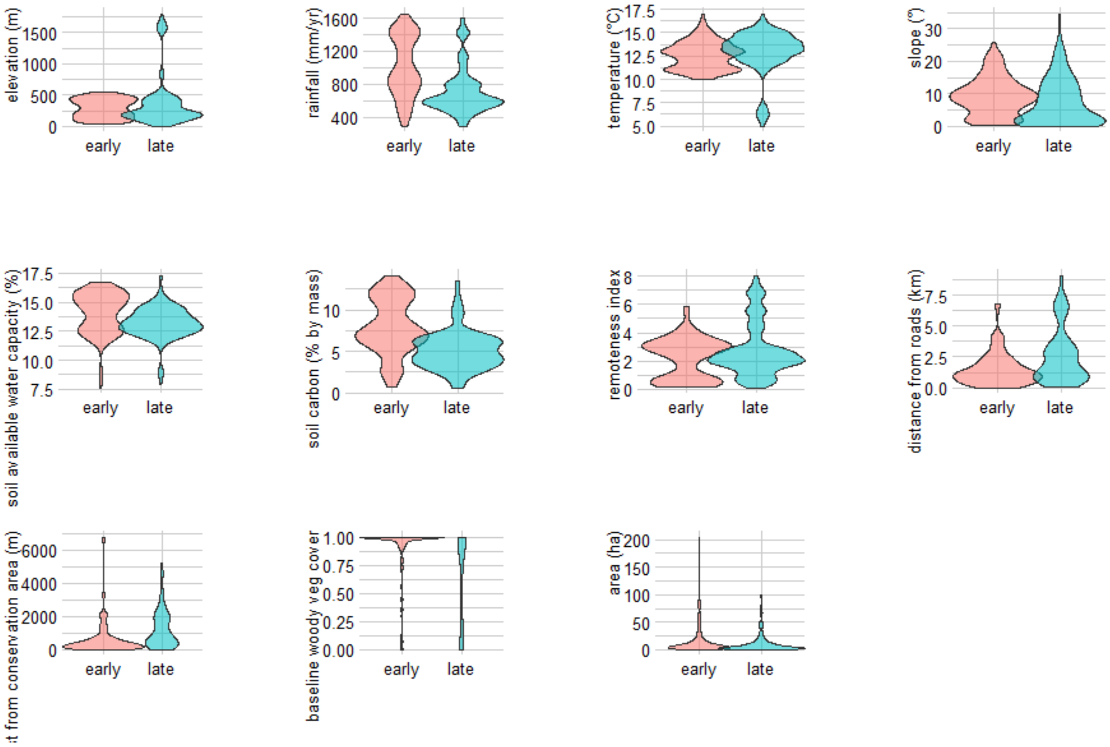

Figure S1. Comparison of the distribution of covariate values between the early (pink) and late offsets (blue) for each covariate.

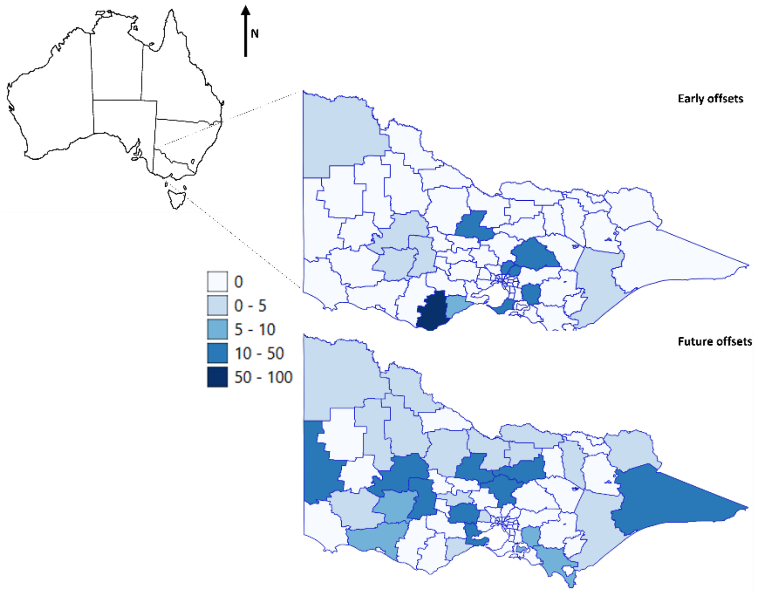

Figure S2. Spatial distribution of early and late offsets by Local Government Area. Darker blue indicates a higher number of offsets and lighter blue indicates a lower number of offsets.

Statistical matching

Smaller standardised mean differences in covariate values between offsets and matched controls indicate better matches, with standardised mean differences <0.1 considered high-quality matches (Greifer 2022). For all specifications, we match 1:1 and without replacement, as our pool of potential controls is vastly larger than treated observations. As a robustness check we conduct two commonly-used matching methods (Sonter et al. 2019; Devenish et al. 2022), and progressively reduce the caliper until there are no further gains in balance or until large numbers of observations are dropped. We use: a) nearest neighbour matching on propensity scores derived using logistic regression; b) Mahalanobis distance matching with exact matching on land use and a caliper of 1 standard deviation; c) and d) the same as b) but with 0.5 and 0.25 standard deviation calipers respectively.

The performance of our alternative matching specifications is detailed in Figures S3 and Figures S4.

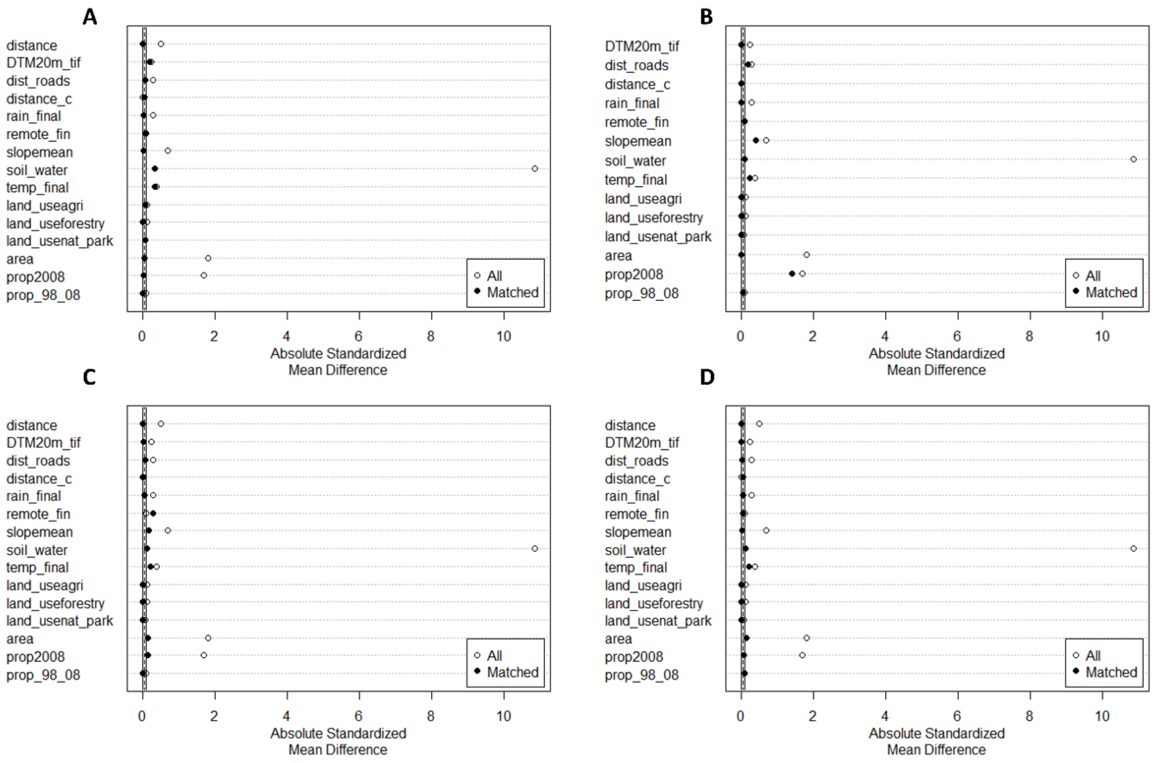

Figure S3. Loveplots showing the standardised mean difference between full and matched datasets and treated observations (regeneration offsets) under various matching specifications. All specifications achieve full matching of treated and control observations: A) 1:1 propensity score matching without replacement; B) 1:1 Mahalanobis distance matching with 1 standard deviation calipers and exact matching for the land use for each land parcel; C) As B, with 0.5 standard deviation calipers; D) As B, with 0.25 standard deviation calipers

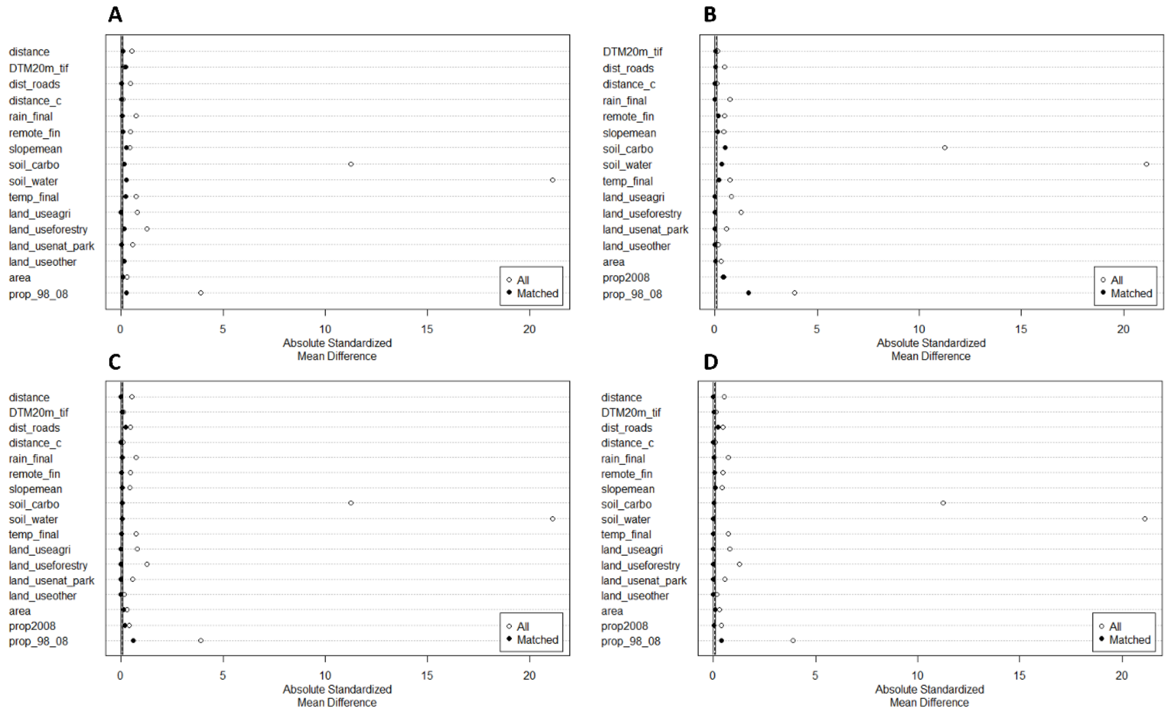

Figure S4. Loveplots showing the standardised mean difference between full and matched datasets and treated observations (avoided loss offsets) under various matching specifications: A) 1:1 propensity score matching without replacement (complete matching of treated and controls); B) 1:1 Mahalanobis distance matching with 1 standard deviation calipers and exact matching for the land use for each land parcel (4 treated observations unmatched and dropped); C) As B, with 0.5 standard deviation calipers (4 treated observations unmatched and dropped); D) As B, with 0.25 standard deviation calipers (4 treated observations unmatched and dropped)

Background trend analysis

To test for parallel trends before implementing the difference-in-difference analysis, we followed the methods of Devenish et al. (2022). We regressed the pre-intervention woody vegetation cover data against the interaction between whether the site is from the control or intervention sample, and year. If the interaction is significant, it implies that there is a significantly different time trend between the offsets and controls. Regression outputs are given in Table S4.

Table S4. Regression outputs for the regressions testing the parallel trends assumptions. Values represent regression coefficients, standard errors in brackets. Significance (p<0.05) is indicated by *

Parameter	Early offsets vs future offsets	Early offsets vs matched non-adopters
Intercept	7.05 (8.48)	4.92
Year	-0.00 (0.00)	-0.00 (0.01)
Treatment	-0.31 (15.27)	0.74 (16.57)
Year:Treatment	0.00 (0.01)	-0.00 (0.01)

Regression outputs

Here we present full outputs of our regression comparing changes in woody vegetation cover between early regeneration offsets and matched controls (Table S5), and early regeneration offsets and late regeneration offsets (Table S6).

*Table S5. Regression outputs for our linear mixed effects model estimating the impact of offset management on woody vegetation cover, comparing regeneration offsets with matched control land parcels. Coefficient estimates and associated standard errors are presented. For the categorical Land use variable, the baseline land use against which alternatives are compared is agriculture. P-values are denoted by stars: *= p<0.05, ***=p<0.001*

**Parameter**	**Coefficient (std errors)**
(Intercept)	0.11 (0.02) ***
Time since policy	-0.00 (0.00)
Before/after intervention dummy	-0.00 (0.02)
Treatment/control dummy	0.06 (0.04)
Baseline woody vegetation cover	0.80 (0.02) ***
Distance from roads	0.02 (0.01)
Elevation	-0.03 (0.02)
Rainfall	0.01 (0.02)
Remoteness	-0.01 (0.02)
Slope	0.01 (0.01)
Temperature	-0.01 (0.03)
Soil water	0.01 (0.01)
Distance from conservation area	-0.04 (0.01) *
Area	-0.01 (0.01)
Land use (conservation area)	0.03 (0.06)
X	-0.02 (0.02)
Y	-0.00 (0.03)
Time since policy: before/after dummy	0.01 (0.00) ***
Time since policy: treatment/control dummy	-0.00 (0.00)
before/after dummy: treatment/control dummy	-0.11 (0.03) ***
Time since policy: bef/aft: treat/control	0.03 (0.00) ***
AIC	-1535.41
Num. obs.	1944
Num. groups: HH_PAI	68
Var: HH_PAI (Intercept)	0.01
Var: Residual	0.02
R^2	0.73

*Table S6. Regression outputs for our linear mixed effects model estimating the impact of offset management on woody vegetation cover, comparing early regeneration offsets with late regeneration offsets. Coefficient estimates and associated standard errors are presented. For the categorical Land use variable, the baseline land use against which alternatives are compared is agriculture. P-values are denoted by stars: *= p<0.05, ***=p<0.001*

Parameter	Coefficient (std errors)
(Intercept)	0.06 (0.02) **
Time since policy	-0.00 (0.00)
Before/after intervention dummy	-0.04 (0.01) **
Treatment/control dummy	0.09 (0.03) **
Baseline woody vegetation cover	0.84 (0.01) ***
Distance from roads	0.01 (0.01)
Elevation	-0.04 (0.02)
Rainfall	-0.01 (0.03)
Remoteness	0.02 (0.02)
Slope	0.00 (0.01)
Temperature	-0.03 (0.02)
Soil water	0.01 (0.01)
Distance from conservation area	-0.03 (0.01) **
Area	-0.01 (0.00) **
Land use (forestry)	0.14 (0.06) *
Land use (conservation area)	-0.08 (0.07)
Land use (other)	0.02 (0.03)
X	-0.00 (0.01)
Y	0.06 (0.02) *
Time since policy: before/after dummy	0.03 (0.00) ***
Time since policy: treatment/control dummy	0.00 (0.00)
before/after dummy: treatment/control dummy	-0.09 (0.02) ***
Time since policy: bef/aft: treat/control	0.01 (0.00) **
AIC	-2456.12
Num. obs.	3150
Num. groups: HH_PAI	52
Var: HH_PAI (Intercept)	0.01
Var: Residual	0.02
R^2	0.80

Sensitivity analyses

Effects of varying the threshold for assigning offsets to 'regeneration' or 'avoided loss'

In our main analysis, we chose the threshold of a proportion native vegetation cover of 0.95 to assign offsets to the regeneration or avoided loss category, because this retains an effective sample size for the regeneration offsets. As we lower the threshold, the sample size declines (0.9, N early offsets=37, total area of regeneration offsets 307 ha; 0.8, N early offsets=29, total area of regeneration offsets 227 ha), and the mean woody vegetation cover in our offsets declines. This means that there is greater potential for woody vegetation cover to increase over the 10-year evaluation period. Therefore, as we reduce the sample size and lower the threshold, the effect size of the impacts of offsets on native vegetation increases slightly (these offsets that start with lower baseline woody vegetation cover experience larger increases in woody vegetation cover than offsets starting with a higher baseline woody vegetation cover).

The regression outputs for the diff-in-diff regression comparing the change in woody vegetation cover in early regeneration offsets and matched non-adopter parcels (using our core model) at varying baseline woody vegetation thresholds is presented in Table S7. When the threshold for regeneration offsets is set at a baseline proportion woody vegetation cover <0.9, early offsets are associated with an increase in woody vegetation cover of 3.09% per year relative to controls, implying that regeneration offsets led to a mid-point additional increase in woody vegetation cover of 95 ha. When the threshold for regeneration offsets is set at a baseline proportion woody vegetation cover <0.8, early offsets are associated with an increase in woody vegetation cover of 4.04% per year relative to controls, implying that regeneration offsets led to a mid-point additional increase in woody vegetation cover of 92 ha.

Table S7. Regression outputs for our linear mixed effects model estimating the impact of offset management on woody vegetation cover, comparing early regeneration offsets with matched non-adopters, and assuming different thresholds for categorising regeneration offsets. Coefficient estimates and associated standard errors are presented. P-values are denoted by stars: *= p<0.05, ***=p<0.001

**Parameter**	**Coefficient (std errors), threshold = 0.9**	**Coefficient (std errors), threshold = 0.8**
(Intercept)	0.08 (0.03) **	0.03 (0.03)
Time since policy	-0.00 (0.00)	-0.00 (0.00)
Before/after intervention dummy	-0.01 (0.02)	0.02 (0.03)
Treatment/control dummy	0.05 (0.04)	0.09 (0.05)
Baseline woody vegetation cover	0.82 (0.03) ***	0.88 (0.03) ***
Distance from roads	0.08 (0.02) ***	0.01 (0.02)
Elevation	-0.01 (0.02)	0.00 (0.04)
Rainfall	0.01 (0.03)	0.02 (0.03)
Remoteness	-0.08 (0.03) **	-0.03 (0.02)
Slope	-0.01 (0.02)	-0.03 (0.02)
Temperature	-0.04 (0.04)	-0.05 (0.05)
Soil water	0.02 (0.02)	0.01 (0.04)
Distance from conservation area	-0.03 (0.02)	-0.03 (0.02)
Area	-0.01 (0.01)	-0.03 (0.01) **
X	-0.02 (0.02)	-0.01 (0.02)
Y	0.02 (0.05)	0.07 (0.06)
Time since policy: before/after dummy	0.02 (0.00) ***	0.01 (0.01) **
Time since policy: treatment/control dummy	0.00 (0.00)	0.00 (0.01)
before/after dummy: treatment/control dummy	-0.12 (0.03) ***	-0.13 (0.04) **
Time since policy: bef/aft: treat/control	0.03 (0.01) ***	0.04 (0.01) ***
AIC	-910.79	-455.64
Num. obs.	1332	972
Num. groups: HH_PAI	50	39
Var: HH_PAI (Intercept)	0.01	0.01
Var: Residual	0.02	0.03
R^2	0.73	0.72

The regression outputs for the diff-in-diff regression comparing the change in woody vegetation cover in early regeneration offsets and future regeneration offsets at varying baseline woody vegetation thresholds is presented in Table S8. When the threshold for regeneration offsets is set at a baseline proportion woody vegetation cover <0.9, early offsets are associated with an increase in woody vegetation cover of 1.85% per year relative to controls, implying that regeneration offsets led to a mid-point additional increase in woody vegetation cover of 57 ha. When the threshold for regeneration offsets is set at a baseline proportion woody vegetation cover <0.8, early offsets are associated with an increase in woody vegetation cover of 2.09% per year relative to controls, implying that regeneration offsets led to a mid-point additional increase in woody vegetation cover of 47 ha.

Table S8. Regression outputs for our linear mixed effects model estimating the impact of offset management on woody vegetation cover, comparing early regeneration offsets with future offsets, and assuming different thresholds for categorising regeneration offsets. Coefficient estimates and associated standard errors are presented. For the categorical Land use variable, the baseline land use against which alternatives are compared is agriculture. P-values are denoted by stars: *= p<0.05, ***=p<0.001

**Parameter**	**Coefficient (std errors), threshold = 0.9**	**Coefficient (std errors), threshold = 0.8**
(Intercept)	0.05 (0.02) *	0.01 (0.02)
Time since policy	-0.00 (0.00) *	-0.01 (0.00) **
Before/after intervention dummy	-0.04 (0.01) **	-0.04 (0.02) *
Treatment/control dummy	0.07 (0.04)	0.09 (0.04) *
Baseline woody vegetation cover	0.88 (0.02) ***	0.94 (0.02) ***
Distance from roads	0.01 (0.01)	0.01 (0.01)
Elevation	-0.03 (0.03)	0.04 (0.03)
Rainfall	-0.02 (0.03)	-0.02 (0.03)
Remoteness	0.02 (0.02)	0.03 (0.03)
Slope	-0.00 (0.01)	-0.01 (0.01)
Temperature	-0.03 (0.03)	0.02 (0.03)
Soil water	0.01 (0.01)	-0.00 (0.02)
Distance from conservation area	-0.03 (0.01) **	-0.03 (0.01) *
Area	-0.02 (0.01) ***	-0.02 (0.01) **
Land use (forestry)	0.14 (0.07) *	0.20 (0.08) *
Land use (conservation area)	-0.21 (0.12)	
Land use (other)	0.00 (0.04)	0.01 (0.04)
X	-0.00 (0.02)	0.00 (0.02)
Y	0.07 (0.03) *	-0.01 (0.04)
Time since policy: before/after dummy	0.03 (0.00) ***	0.04 (0.00) ***
Time since policy: treatment/control dummy	0.00 (0.00)	0.00 (0.00)
before/after dummy: treatment/control dummy	-0.10 (0.03) ***	-0.10 (0.03) **
Time since policy: bef/aft: treat/control	0.02 (0.00) ***	0.02 (0.01) ***
AIC	-1721.09	-1252.34
Num. obs.	2538	2070
Num. groups: HH_PAI	50	45
Var: HH_PAI (Intercept)	0.01	0.01
Var: Residual	0.03	0.03
R^2	0.77	0.72

Effects of removing sites burned by wildfires during the analysis period

Removing the landholding containing offsets which experienced catastrophic loss of woody vegetation cover in the 2009 Black Saturday fires altered the outputs of the regression models, as the rapid vegetation regrowth in these offsets caused by the fire but coincident with the onset of offset management in 2008 contributed in increasing the effect size of offset management. Full results of the core regression analyses excluding these offsets is in Table S9.

Table S9. Regression outputs for our linear mixed effects models estimating the impact of offset management on woody vegetation cover, excluding sites burned by wildfires. Coefficient estimates and associated standard errors are presented. P-values are denoted by stars: *= p<0.05, ***=p<0.001

**Parameter**	**Coefficient (std errors), early offsets versus matched non-adopters**	**Coefficient (std errors), early offsets versus future offsets**
(Intercept)	0.14 (0.03) ***	0.06 (0.02) ***
Time since policy	0.00 (0.00)	-0.00 (0.00)
Before/after intervention dummy	-0.03 (0.02)	-0.04 (0.01) **
Treatment/control dummy	0.02 (0.04)	0.09 (0.03) **
Baseline woody vegetation cover	0.79 (0.02) ***	0.84 (0.01) ***
Distance from roads	0.05 (0.02) **	0.01 (0.01)
Elevation	-0.01 (0.02)	-0.03 (0.03)
Rainfall	-0.01 (0.03)	-0.02 (0.03)
Remoteness	-0.01 (0.02)	0.01 (0.02)
Slope	-0.01 (0.01)	0.00 (0.01)
Temperature	-0.03 (0.03)	-0.03 (0.02)
Soil water	-0.00 (0.02)	0.00 (0.01)
Distance from conservation area	-0.06 (0.02) ***	-0.03 (0.01) ***
Area	-0.01 (0.01)	-0.01 (0.00) **
X	-0.01 (0.02)	-0.00 (0.01)
Y	-0.01 (0.04)	0.05 (0.02) *
Time since policy: before/after dummy	0.01 (0.00) **	0.03 (0.00) ***
Time since policy: treatment/control dummy	-0.00 (0.00)	0.00 (0.00)
before/after dummy: treatment/control dummy	-0.04 (0.03)	-0.04 (0.02)
Time since policy: bef/aft: treat/control	0.02 (0.00) ***	0.01 (0.00)
AIC	-1664.57	-2603.80
Num. obs.	1692	3060
Num. groups: HH_PAI	61	51
Var: HH_PAI (Intercept)	0.01	0.01
Var: Residual	0.02	0.02
R^2	0.77	0.72
